# Draft Genome Sequence of the Hydrogen- and Ethanol-Producing Bacterium *Clostridium intestinale* Strain URNW

**DOI:** 10.1128/genomeA.00871-13

**Published:** 2013-10-17

**Authors:** Sadhana Lal, Umesh Ramachandran, Xiangli Zhang, Richard Sparling, David B. Levin

**Affiliations:** Department of Biosystems Engineering, University of Manitoba, Winnipeg, Manitoba, Canadaa; Department of Microbiology, University of Manitoba, Winnipeg, Manitoba, Canadab; Department of Plant Science, University of Manitoba, Winnipeg, Manitoba, Canadac

## Abstract

Here, we report the draft genome sequence of *Clostridium intestinale* strain URNW, which can convert biomass to useful products such as biofuels (hydrogen or ethanol) and other soluble end products.

## GENOME ANNOUNCEMENT

*Clostridium intestinale* strain URNW is a Gram-positive, mesophilic, anaerobic spore-forming bacterium (1). Its optimum growth temperature is 37°C, but it can grow at a range of temperatures from 22°C to a maximum of 45°C and has displayed a doubling time of 1.5 h (1). Nucleotide sequence analyses of *Clostridium intestinale* URNW 16S rRNA genes and chaperonin 60 (*cpn*60) genes revealed a 98% identity with *C. intestinale* ATCC 49213 (1, 2). Phylogenetic analyses based on 16S rDNA and *cpn*60 nucleotide sequences indicated that *C. intestinale* URNW clustered with butyrate-producing hydrogen producers, such as *C. intestinale*, *C. acetobutylicum*, *C. perfringens*, *C. butyricum*, and *C. beijirinckii* (1).

The genome of *C. intestinale* strain URNW was sequenced by the McGill University and Genome Quebec Innovation Centre by use of the paired-end and shotgun protocols of the Roche/454s GS-FLX Titanium system and the paired-end protocol of the Illumina Hiseq 2000 platform. The raw reads of the 454 and Illumina were first preprocessed with GS2.6 and Trimmomatic-0.22 (3) for adapter clipping and quality trimming before assembly. After preprocessing, mixed paired-end and unpaired Illumina short reads and 454 long reads were pooled for assembly with the Velvet (4) assembler. Following Velvet assembly, further scaffolding and gap filling were performed with SSPACE v2.0 (5) and GapFiller v 1.11 (6). The final draft genome assembly has coverages of ~600 for Illumina reads and ~28 for 454 reads and contains 39 contigs with a total size of 4,671,178 bp, an *N*_50_ contig length of 351,514 nucleotides, and a mean G+C content of 30.2%. The average contig length of is 119,774 bp and the longest contig length is 1,533,141 bp.

The draft genome assembly was submitted to NCBI WGS genome sequencing project and annotated with the NCBI Prokaryotic Genome Annotation Pipeline (PGAP). The NCBI PGAP annotation GenBank file was uploaded to the web service of the Gene Prediction Improvement Pipeline (GenePRIMP) (7) developed by JGI for analysis of gene anomalies. Based on the report of GenePRIMP anomalies, manual curation was done using Artemis (8). The final annotation of the draft genome sequence of *C. intestinale* strain URNW has a total of 4,660 genes, including 4,367 protein-coding genes, 182 pseudogenes, 83 tRNAs, and 28 rRNAs.

*C. intestinale* strain URNW protein-coding genes were verified using other *Clostridium* species as reference organisms. Amino acid sequences for each gene product were retrieved from the JGI genome portal (http://genome.jgi-psf.org/) and the NCBI database (http://www.ncbi.nlm.nih.gov/genomes/lproks.cgi), and sequence alignments against *C. intestinale* strain URNW genes were performed. Key enzymes involved in core metabolism were identified using an *in silico* approach, which revealed that *C. intestinale* strain URNW is a potential candidate for the production of biofuels such as hydrogen or ethanol. All 39 contigs were analyzed by the online software IslandViewer and did not show any genes associated with pathogenicity (9).

### Nucleotide sequence accession numbers.

The genome sequence of *Clostridium intestinale* strain URNW has been deposited at DDBJ/EMBL/GenBank under the accession no. APJA00000000. The version described in this paper is the first version, accession no. APJA01000000.
